# Antiapoptosis and Antifibrosis Effects of *Qishen* Granules on Heart Failure Rats via Hippo Pathway

**DOI:** 10.1155/2019/1642575

**Published:** 2019-12-12

**Authors:** Jian Zhang, Junjie Liu, Sheng Gao, Weili Lin, Pengrong Gao, Kuo Gao, Yili Zhang, Kangjia Du, Xiaomin Yang, Wei Wang, Ruixin Zhu, Yong Wang

**Affiliations:** ^1^School of Life Science, Beijing University of Chinese Medicine, Beijing 100029, China; ^2^Dongzhimen Hospital, Beijing University of Chinese Medicine, Beijing 100700, China; ^3^School of Life Sciences and Technology, Tongji University, Shanghai 200092, China; ^4^School of Traditional Chinese Medicine, Beijing University of Chinese Medicine, Beijing 100029, China

## Abstract

*Qishen* granules (QSG) are a famous formula with cardioprotective properties to heart failure (HF). The aim of this study was to investigate the underlying mechanism of QSG on apoptosis and fibrosis in the treatment of HF. HF model was induced by left anterior descending artery ligation on Sprague-Dawley rats. Transcriptome analysis was used to investigate the regulatory pathways of QSG on HF. Interestingly, downregulated genes of QSG were significantly enriched in Hippo pathway which plays a crucial role in regulating cell apoptosis and proliferation. We found that QSG inhibited the expressions of proapoptotic key proteins P-53 and fibrosis-related proteins TGF-*β*1, SMAD3, and CTGF. Further, we conducted research on the key proteins in the Hippo pathway upstream of CTGF and P-53. The results showed that MST1, P-MST1, P-LATS1, and RASSF1A that exert proapoptotic function were downregulated after QSG intervention. Similarly, P-YAP and P-TAZ, mediating self-degradation and apoptosis, were both observably decreased after QSG administration. Taken together, QSG are shown to be likely to exert cardioprotective effects by inhibiting the progression of apoptosis and fibrosis through Hippo pathway.

## 1. Introduction

Heart failure (HF) imposes a major global health care burden on society and suffering on the individual [1, 2]. Although surgical and medical advances over decades have greatly improved survival after HF, the long-term prognosis of these patients remains unsatisfying [3]. Therefore, it is urgently needed to explore intrinsic mechanism of HF and new strategies for HF. Previous studies have shown that apoptosis and fibrosis are the main pathological mechanisms of HF [4, 5]. Consequently, anticardiomyocyte apoptosis and reversing fibrosis are the most attractive strategies for HF treatment [6].

The Hippo pathway, originally identified in drosophila as an important regulator of cardiomyocyte apoptosis and proliferation [7], has attracted recent interest as a potential approach to treat HF [8–10]. Mammalian sterile 20-like kinase 1/2 (MST1/2), the chief component of the Hippo pathway [11, 12], can translocate to mitochondria and phosphorylate B-cell lymphoma-2 (Bcl-2), causing Bcl-2-associated X protein (Bax) activation with a subsequence of cardiomyocyte apoptosis during myocardial ischemic injury [13]. Meanwhile, MST1/2 can activate large tumor suppressor kinase 1/2 (LATS1/2) thus to activate P53 or phosphorylates yes-associated protein (YAP) and transcriptional coactivator with PDZ-binding motif (TAZ), which can also contribute to apoptosis [14]. Of note, YAP/TAZ can initiate the transcription of connective tissue growth factor (CTGF) that mediates the expressions of extracellular matrix genes and promotes cardiac remodeling and fibrosis by interacting with TEA domain (TEAD) family and other transcription factors, such as TGF-*β*-induced SMAD2/3 [8, 15–17]. Therefore, Hippo pathway can be a potential therapeutic target for reducing cardiomyocyte apoptosis and treating myocardial fibrosis in HF.

*Qishen* granules (QSG), composed of Radix Astragali, Radix Salvia Miltiorrhizae, Flos Lonicerae, Radix Scrophulariae, Radix Aconiti Lateralis Preparata, and Radix Glycyrrhizae (Table 1), are a famous formula with cardioprotective properties prescribed to HF for many years. The previous studies have demonstrated that QSG could inhibit myocardial inflammation injury, cardiomyocyte apoptosis, and myocardial fibrosis [18–21] while its underlying mechanism remains to be further defined. Transcriptomics is a potent tool that offers convenient and reliable access to changes in the expression of genes, which could shed light into multiplexed mechanisms of drugs [22]. In this study, mRNA transcriptomic analysis was firstly used to investigate the regulatory pathway of QSG on HF rat model. Interestingly, transcriptomic analysis results indicated that QSG could prevent HF by regulating Hippo pathway. Given that the Hippo pathway plays a crucial role in regulating cell apoptosis and proliferation, we investigated the regulatory mechanism of QSG on apoptosis and fibrosis effects via Hippo pathway in the prevention of HF.

## 2. Materials and Methods

### 2.1. Experimental Animals

Male Sprague-Dawley rats with weights of 240 ± 10 g were obtained from the Vital River Laboratory Animal Technology Co. Ltd. (Beijing, China). The room in which rats were housed was 21 ± 2°C and 55 ± 5% relative humidity with artificial 12 : 12 hours equivalent light-dark cycles. All experimental procedures were carried out according to the National Institute of Health Guide for the Care and Use of Laboratory Animals and approved by the Animal Care Committee of Beijing University of Chinese Medicine.

### 2.2. Preparation and Quantitative Analysis of QSG

QSG consists of 6 Chinese herbs, that is, Radix Astragali, Radix Salvia Miltiorrhizae, Flos Lonicerae, Radix Scrophulariae, Radix Aconiti Lateralis Preparata, and Radix Glycyrrhizae. Herbal sources, preparation method, and quantitative analysis of QSG were stated in detail in our previous study [21]. The same batch of QSG was used for all the experiments in this study.

### 2.3. Animal Grouping, HF Model Induction, and Drug Administration

Total 60 rats were randomly divided into the sham group, model group, QSG group, and fosinopril group (15 rats per group) using a computer-generated random number table. Rats in model, QSG, and fosinopril groups received ligation surgery of left anterior descending coronary artery as previously reported [23]. Briefly, left thoracotomy between the third and fourth intercostal spaces was performed on rats. After exposing the cardiac tissues, left anterior descending coronary artery was ligated with a sterile suture (Shuangjian, Shanghai, China) 1 mm below the left atrium. The thorax was then closed layer by layer. Sham-operated rats were manipulated in the same way with no actual ligation of left anterior descending coronary artery.

QSG and fosinopril (Bristol-Myers Squibb, China) were dissolved in sterile saline. The rats in the QSG group were treated with QSG at a daily dose of 18.66 g/kg for 28 days as previous study [21]. The rats in the positive control group were treated with fosinopril at a daily dose of 1.2 mg/kg as in a previous study [21]. Rats in the sham group and model group were given normal saline (10 ml/kg/day).

There was no animal death in the sham group during the entire experiment, while the mortality rate of rats in the model group, QSG group, and fosinopril group was 26.7%, 20.0%, and 13.3% during the entire experiment, respectively.

### 2.4. Assessment of Cardiac Functions by Echocardiography

Echocardiography was applied to detect the LVESD, LVEDD, EF, and FS. A PST 65A sector scanner (8 MHz probe) was employed, which generates two-dimensional images at a frame rate of 300 to 500 frames/s. The left ventricular dimension was measured using M-model fractional shortening, and FS was calculated using the following equation: FS = [(LVEDD − LVESD)/LVEDD] × 100%. EF was calculated using the following equation: EF = [(LVEDV − LVESV)/LVEDV] × 100%.

### 2.5. Measurement of Serum Biochemical Markers

At the end of the cardiac functions examination, rats were anesthetized with 1% pentobarbital sodium (50 mg/kg) by intraperitoneal injection, and blood samples were collected from the abdominal aorta and centrifuged at 1000 ×g for 20 min to obtain serum. Blood samples were collected and processed to serum. Serum levels of ALD, BNP, ANP, PIIINP, MMP-2, and MMP-9 were measured by automatic biochemical analyzer (HITACH17080, Tokyo, Japan) following the instructions of kits (Sekisui Chemical Company, Tokyo, Japan).

### 2.6. Hematoxylin and Eosin Staining

The hearts (*n* = 4) were excised and irrigated with saline solution and fixed in 4% paraformaldehyde solution for more than 48 h and embedded in paraffin. Sections were cut 5 mm thick for further histological analysis. Sections were stained with hematoxylin and eosin staining to visualize cardiomyocyte architecture. Sections were deparaffinized by immersion in xylene and stained with hematoxylin for 5 min. Sections were washed in double-distilled water three times and placed in 85% alcohol for 2 min. The sections were stained with eosin for 5 min and incubated in an ascending alcohol concentration gradient (70, 80, and 90%) for 5 min. Sections were placed in 100% alcohol for 5 min and twice in xylene for 1 min. Images were visualized under an optical OLYMPUS microscope.

### 2.7. Masson's Trichrome Staining

Tissue sections were deparaffinized via immersion in xylene (3 times, 5 min each) and rehydrated using a descending series of alcohols (100%, 90%, 85%, and 75% alcohol, 5 min each). Biopsy samples were stained using Masson's trichrome staining to investigate heart morphological and fibrotic changes. Blue staining represented collagen accumulation. Images were visualized under an optical OLYMPUS microscope. Fibrotic areas fraction was calculated using Image-Pro Plus software after Masson's staining. Fibrotic areas fraction = (area of collagen)/(area of visual field) × 100%.

### 2.8. TUNEL Staining

TUNEL (transferase-mediated deoxyuridine triphosphate-biotin nick end labeling) analysis of tissue sections was performed using Roche fluorescein TUNEL kit (11684817910, Roche, Mannheim, Germany) to assess apoptosis following the manufacturer's instructions and observed by fluorescence microscope (C1 Digital Eclipse, Nikon, Tokyo, Japan). Apoptotic nuclei were labeled with green fluorescein staining and total cardiomyocyte nuclei were marked with DAPI. The ratio of TUNEL-positive cell to all area was calculated by Image-Pro Plus 6.0.

### 2.9. Transcriptome Profile Analysis and Differential Expression Analysis

RNAs were extracted from the infarct border zone of cardiac tissues by TRIzol Reagent (Gibco-BRL, Paisley, UK), then purification was performed, and mRNAs were reverse transcribed into cDNA using oligo(dT) magnetic beads. The details of linear PCR amplification and sequencing of samples are described in the article. Dirty tags were filtered and then we aligned all clean tags with rat reference sequences. The number of clean tags was normalized (TPM normalized). Differentially expressed genes (DEGs) were identified by NOIseq R package with FDR < 0.05. Differentially expressed proteins (DEPs) were detected by *t*-test and *P* value were adjusted with FDR < 0.05.

### 2.10. Western Blotting

Proteins were extracted from the left ventricular peri-infarct tissues, using RIPA buffer (50 mM Tris-HCl pH 7.4, 150 mM NaCl, 1% NP-40, and 0.1% SDS) containing a protease inhibitor cocktail (Sigma, St. Louis, MO, USA). Equal amounts of protein were subjected to SDS-PAGE and transferred onto PVDF membranes. Standard Western blot analysis was conducted using P-MST1 (1 : 2000 dilution; Cell Signaling: #3681). Glyceraldehyde 3-phosphate dehydrogenase antibody (1 : 10,000 dilution, Cell Signaling Technology: 5174s) was used as a loading control. After incubation with the appropriate secondary antibodies, signals were visualized using the ECL Plus Western blotting detection reagents for 1 min at room temperature. The bands in the membrane were visualized and densitometric analysis of band intensity was performed using Imagelab software (Bio-Rad, Hercules, CA, USA). Then, the same procedure was taken to detect the P53 (1 : 2000 dilution; Abcam: ab26), P-TAZ (1 : 2000 dilution; Cell Signaling: #59971), CTGF (1 : 2000 dilution; Santa Cruz Biotechnology: sc-101586), P-MST1 (1 : 2000 dilution; Cell Signaling: #3681), P-LATS1 (1 : 2000 dilution; Cell Signaling: #9157), SMAD3 (1 : 2000 dilution; Cell Signaling: C67H9), RASSF1 (1 : 2000 dilution; Abcam: ab180801), TGF-beta (1 : 2000 dilution; Cell Signaling: 3711S), P-YAP (1 : 2000 dilution; Cell Signaling: 4911S), YAP (1 : 2000 dilution; Abcam: ab39361), and TAZ (1 : 2000 dilution; Abcam: ab224239).

### 2.11. Statistical Analysis

Data are expressed as means ±standard error (SE). ANOVA using SAS 9.2 statistical software (SAS Institute, NC, USA) was applied to evaluate between-group differences in the outcome variables; follow-up least significant differences analysis verified that these differences were significant. *P* < 0.05 was considered statistically significant. A significant difference was considered if the *P* value was less than 0.05.

## 3. Results

### 3.1. QSG Improved Cardiac Function in HF

**Figure 1:**
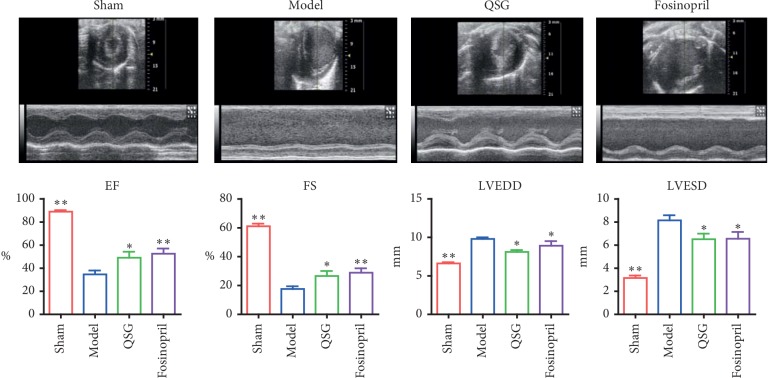
Assessments of cardiac function by echocardiography (*n* = 10 per group). Bars are represented as mean ± SE. Statistically different from the model group: ^*∗*^*P* < 0.05, ^*∗∗*^*P* < 0.01.

Twenty-eight days after surgery, echocardiography showed downregulation of ejection fraction (EF) and fractional shortening (FS) in the model group as compared with the sham group (*P* < 0.01, Figure 1), indicating the cardiac dysfunction in HF. This was accompanied by the enlargement of left ventricular end-diastolic diameter (LVEDD) and left ventricular end-systolic diameter (LVESD) (*P* < 0.01, Figure 1). In contrast, EF and FS increased obviously while LVEDD and LVESD decreased significantly in the QSG and fosinopril groups compared to the model group (*P* < 0.01 or *P* < 0.05, Figure 1), suggesting that QSG could improve cardiac functions in HF.

### 3.2. QSG Reduced Histopathological Damage in HF

**Figure 2:**
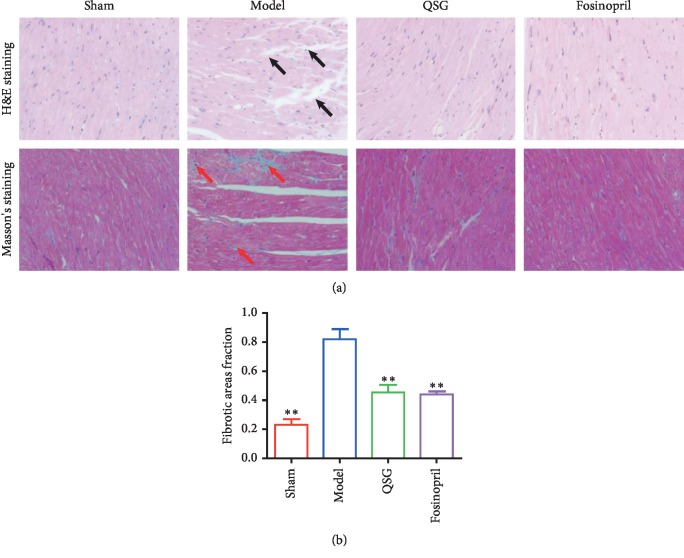
(a) Myocardial samples of the four groups surrounding an area of infarction visualized via H&E staining and (400x) and Masson's staining (400x). Inflammatory cell infiltration is indicated by black arrows. Collagen accumulation is shown in red arrows. (b) Fibrotic areas fraction. *n* = 4 per group. Bars are represented as mean ± SE. Statistically different from the model group: ^*∗*^*P* < 0.05, ^*∗∗*^*P* < 0.01.

Hematoxylin and eosin (H&E) staining (Figure 2(a)) showed that cardiomyocytes in the sham group were orderly arranged, and the nuclei were lightly stained. The myocardial tissue in the model group exhibited obvious pathological abnormalities with pyknotic dark-staining nuclei and inflammatory cell infiltration. Masson's staining (Figure 2(a)) was used to observe the degree of myocardial fibrosis. Normal myocardial tissue was red, and the blue fibrin component appeared in the myocardial interstitium in the model group. The cellular degeneration, inflammatory cell infiltration, and collagen deposition were improved in the QSG and fosinopril groups compared with those in the model group. The fibrotic areas fraction in the model group were upregulated compared with the sham group (*P* < 0.01, Figure 2(b)). The fibrotic areas fraction in the QSG and fosinopril groups decreased significantly compared with the model group (*P* < 0.01, Figure 2(b)).

**Figure 3:**
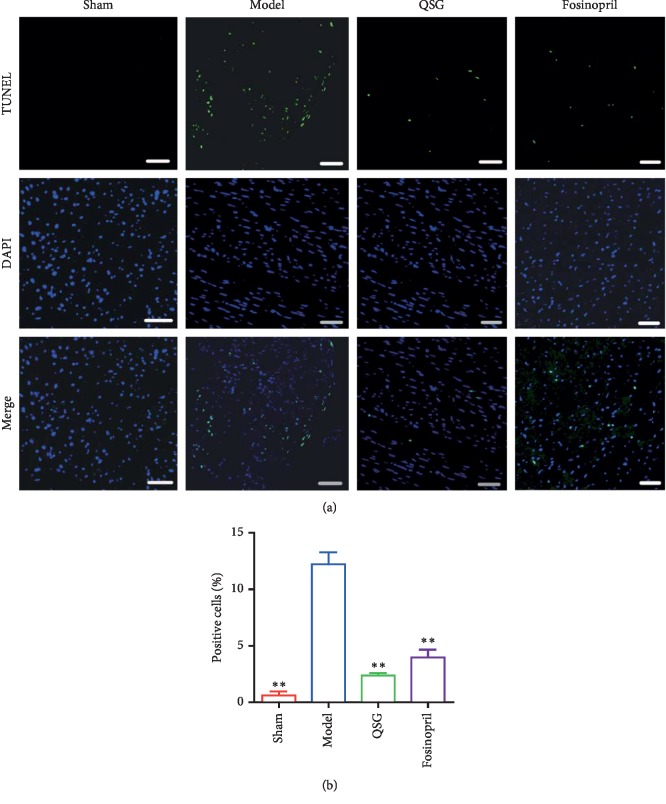
(a) Representative TUNEL staining (200x) of the four groups. TUNEL-positive cells are shown in green fluorescence. DAPI-counterstained nuclei are shown in blue fluorescence. Scale bar: 50 *μ*m. (b) Statistical analysis of the positive cells from each group. *n* = 4 per group. Values are means ± SE. ^*∗*^*P* < 0.05, ^*∗∗*^*P* < 0.01 versus the model group.

TUNEL examination showed that there was almost no apoptosis among cardiac myocytes in the sham group, while apoptosis was observed in the model group, QSG group, and fosinopril group (Figure 3(a)). Evaluation of the positive cells from each group indicated that apoptosis was significantly induced in the model group compared with the sham group (*P* < 0.01, Figure 3(b)). However, after treatment of QSG and fosinopril, apoptosis was markedly reduced (*P* < 0.01, Figure 3(b)).

### 3.3. QSG Reduced ANP and BNP Levels in Serum

**Figure 4:**
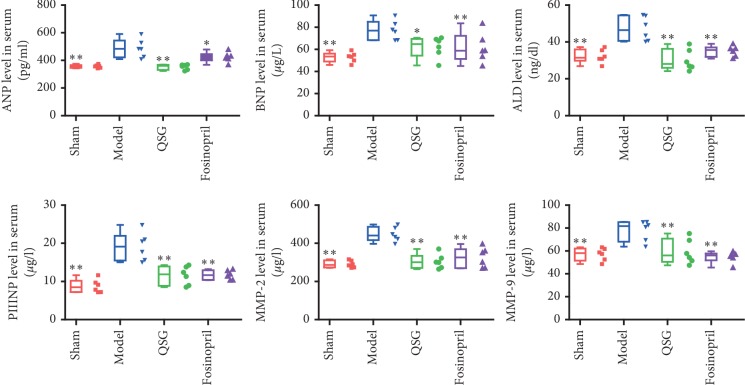
The expression levels of ANP, BNP, ALD, PIIINP, MMP-2, and MMP-9 in serum in the four groups. *n* = 6 per group. Values are means ± SE. Asterisks indicate significant differences. ^*∗*^*P* < 0.05, ^*∗∗*^*P* < 0.01 versus the model group.

Atrial natriuretic peptide (ANP) and brain natriuretic peptide (BNP) are sensitive biomarkers of cardiac dysfunction [24]. In this study, ANP and BNP levels were markedly elevated in the model group compared with the sham group (*P* < 0.01). After 28 days of drug administration, ANP and BNP levels decreased significantly compared with the model group (*P* < 0.01 or *P* < 0.05, Figure 4).

### 3.4. QSG Reduced ALD, PIIINP, MMP-2, and MMP-9 Levels in Serum

Aldosterone (ALD) has many tissue effects including stimulating the production of reactive oxygen species, inflammation, and fibrosis of the heart [25, 26]. In this study, ALD level of model group was markedly elevated compared with the sham group (*P* < 0.01, Figure 4). After QSG administration, a significant reduction of ALD was observed compared with the model group (*P* < 0.01, Figure 4). Procollagen type III N-terminal peptide (PIIINP) has been shown to be serum marker highly associated with myocardial fibrosis [27, 28]. A steady increase of PIIINP was observed in the model group compared with the sham group (*P* < 0.01, Figure 4). However, this change was reversed by QSG treatment (*P* < 0.01, Figure 4). Matrix metalloproteinase-2 (MMP-2) and matrix metalloproteinase-9 (MMP-9) are important for degradation of the extracellular matrix [29]. In this study, MMP-2 and MMP-9 levels in serum were significantly upregulated in the model group compared with the sham group (*P* < 0.01, Figure 4). Following the treatment with QSG, MMP-2 and MMP-9 levels were downregulated (*P* < 0.01, Figure 4).

### 3.5. Transcriptome Expression Profiles and Differential Expression Analysis

**Figure 5:**
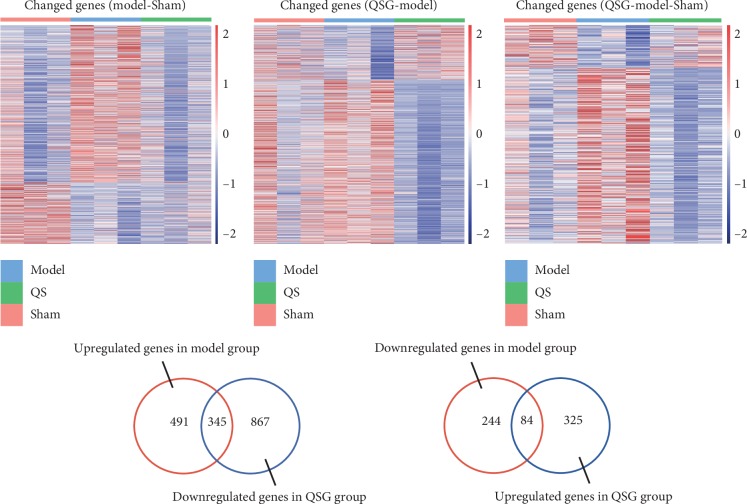
Differentially expressed genes between groups were shown in the heat map and the number of differentially expressed genes between groups.

Differentially expressed genes (DEGs) between groups were shown in heat map (Figure 5). RNA-seq analysis identified 1164 DEGs between model and sham groups, including 836 upregulated DEGs ([Supplementary-material supplementary-material-1]) and 328 downregulated DEGs ([Supplementary-material supplementary-material-1]). The 1621 DEGs between QSG treated group and model group were identified, including 409 upregulated DEGs ([Supplementary-material supplementary-material-1]) and 1212 downregulated DEGs ([Supplementary-material supplementary-material-1]). 345 upregulated and 84 downregulated HF pathogenic genes were regulated inversely in QSG treated group (Figure 5).

### 3.6. Differential Expression Gene-Based Pathway Analysis

**Figure 6:**
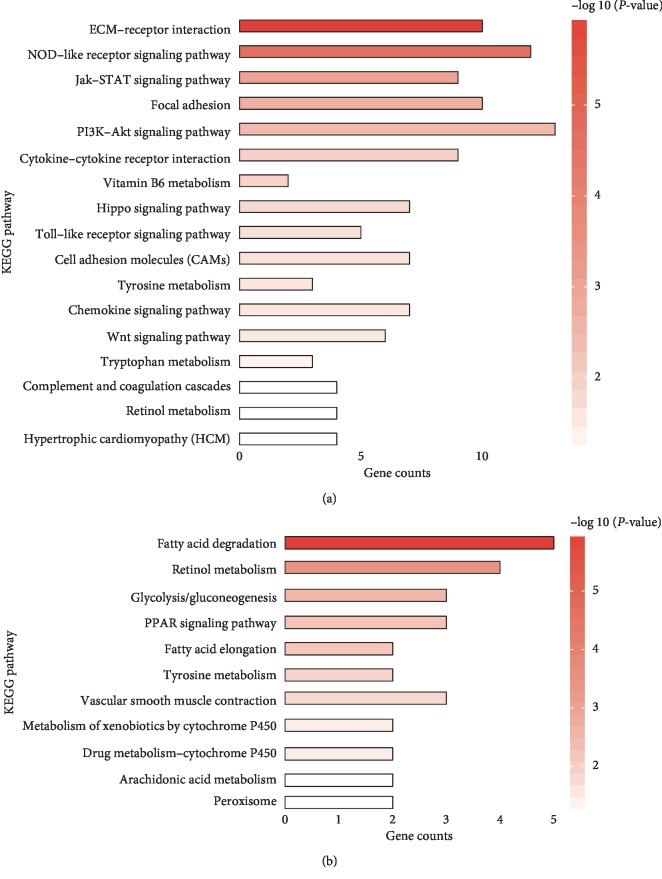
KEGG pathways enriched by inversely downregulated DEGs (a) and inversely upregulated DEGs (b) and they are ordered by −log 10 (*P* value) decreasingly.

Pathway enrichment analysis indicated that the inversely downregulated genes by QSG were mainly enriched in myocardial fibrosis, apoptosis, and inflammation related pathways, such as Hippo, ECM-receptor interaction, focal adhesion, Wnt, PI3K-AKT, chemokine, JAK-STAT, NF-*κ*B, and TNF signaling pathways (Figure 6(a)); the inversely upregulated genes by QSG were mainly enriched in energy metabolism related pathways, such as PPAR, fatty acid metabolism, and fatty acid elongation signaling pathways (Figure 6(b)).

### 3.7. QSG Regulated Myocardial Fibrosis-Related and Apoptosis-Related Proteins

**Figure 7:**
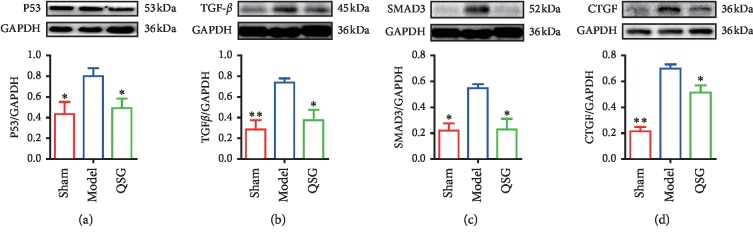
Myocardial protein expression levels of P53, TGF-*β*, SMAD3, and CTGF in the three groups (*n* = 6 per group). Values are means ± SE. Asterisks indicate significant differences. ^*∗*^*P* < 0.05, ^*∗∗*^*P* < 0.01 versus the model group.

As the most classical signaling pathway for regulating fibrosis, the TGF-*β* pathway was included in the study to further investigate the mechanism of QSG antifibrosis [30]. The expressions of TGF-*β*1 and SMAD3 were significantly upregulated in the HF model group compared with the sham group at the endpoint of 28 days (*P* < 0.01 or *P* < 0.05, Figure 7). However, after the intervention of QSG, the levels of TGF-*β*1 and SMAD3 were downregulated (*P* < 0.05, Figure 7). More importantly, CTGF, the downstream target of Hippo pathway which induces the synthesis and secretion of ECM proteins [31], was significantly increased in HF rats (*P* < 0.01, Figure 7). QSG significantly reduced its expressional level compared with the model group (*P* < 0.05, Figure 7).

In addition, the upregulation of proapoptotic protein P-53 was detected in HF (*P* < 0.05, Figure 7). Treatment with QSG significantly inhibited the expressions of P-53 (*P* < 0.05, Figure 7).

### 3.8. QSG Regulated Key Molecules Involved in the Hippo Pathway

**Figure 8:**
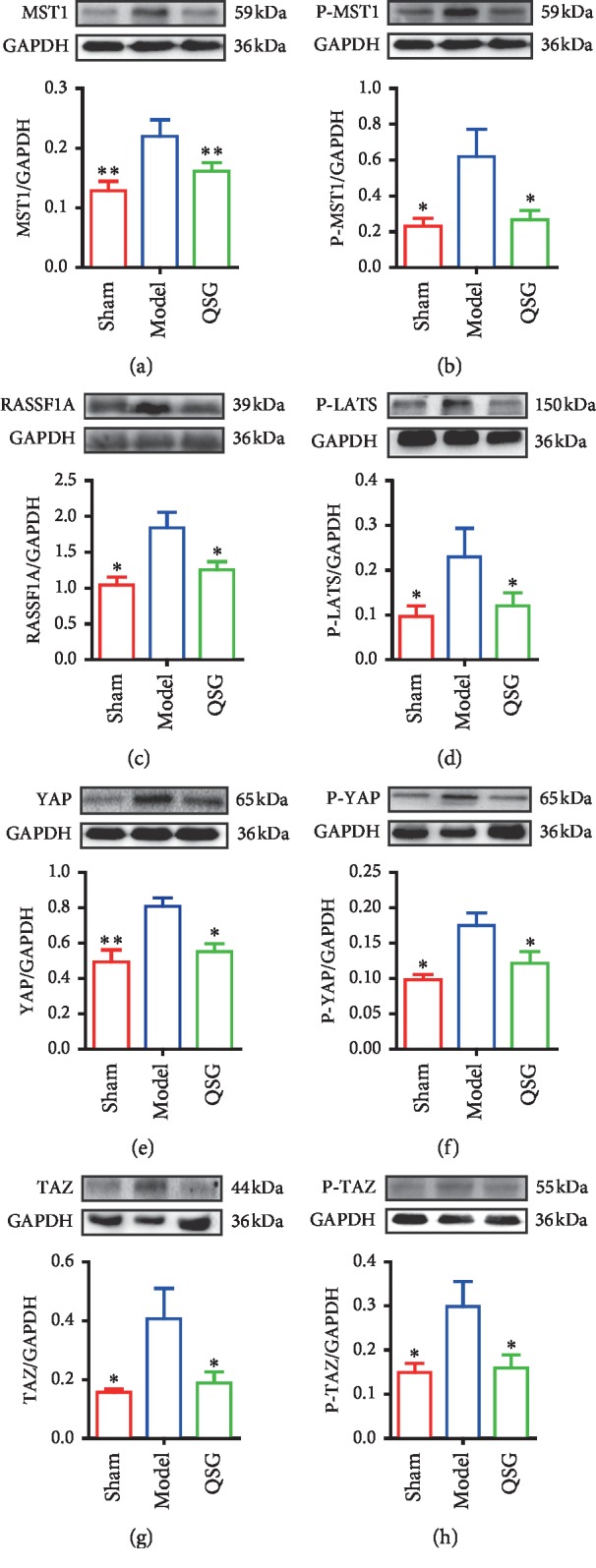
Myocardial protein expression levels of MST1, P-MST1, RASSF1A, P-LATS, YAP, P-YAP, TAZ, and P-TAZ in the three groups (*n* = 6 per group). Values are means ± SE. Asterisks indicate significant differences. ^*∗*^*P* < 0.05, ^*∗∗*^*P* < 0.01 versus the model group.

In the model group, MST1, which is the crucial component of the Hippo pathway and exerts proapoptotic function, was upregulated versus the sham group (*P* < 0.01, Figure 8). Similarly, P-MST1 was found to be elevated in rats of HF (*P* < 0.05, Figure 8). Besides recombinant human ras association domain-containing protein 1 (RASSF1A), the upstream molecule which can stimulate MST1 was upregulated in the model group as well (*P* < 0.05, Figure 8). P-LATS1 was also elevated in HF model (*P* < 0.05, Figure 8). However, these increases returned to the similar levels as in the sham group after QSG treatment (*P* < 0.01 or *P* < 0.05, Figure 8). YAP and its coactivator TAZ, the main downstream effectors of the Hippo pathway regulating proliferation [32], were both markedly increased in HF rats compared with the sham group (*P* < 0.01 or *P* < 0.05, Figure 8). Meanwhile, P-YAP and P-TAZ, mediating self-degradation and apoptosis, were also elevated in HF model rats compared with the sham group (*P* < 0.05, Figure 8). However, the protein levels of YAP, P-YAP, TAZ, and P-TAZ were shown to be significantly decreased by QSG (*P* < 0.05, Figure 8).

## 4. Discussion

**Figure 9:**
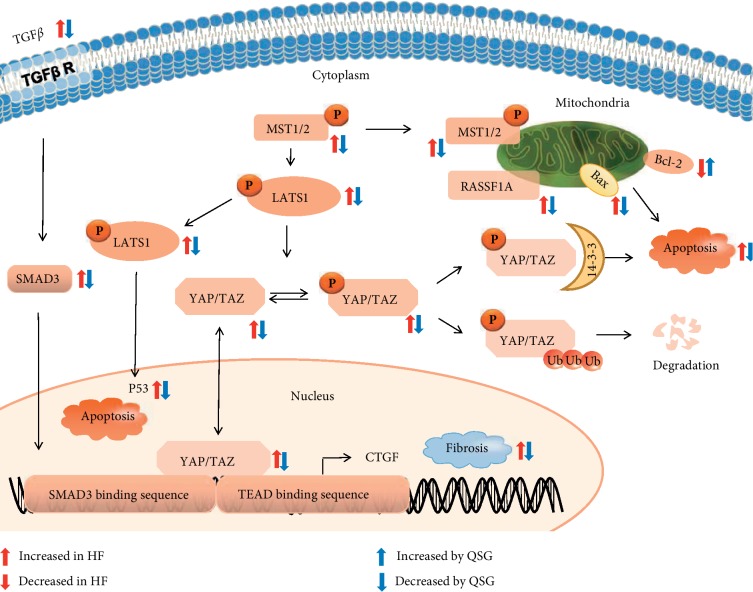
A schematic showing the cardioprotective effects of QSG by inhibiting the progression of apoptosis and fibrosis through Hippo pathway.

During the process of HF, the billions of cardiomyocytes lost cannot be restored, being replaced by fibrotic myocardial tissue with little contractile function and poor diastolic compliance [33]. Therefore, anticardiomyocyte apoptosis and antimyocardial fibrosis have become the key to treating HF. The Hippo pathway plays an important role in regulating cell apoptosis and proliferation. Hence, targeting key molecules in the Hippo pathway could offer an alternative therapeutic approach in the management of cardiomyocyte apoptosis and myocardial fibrosis. In our study, first, transcriptomic study indicated that QSG could regulate Hippo pathway against HF. Then, QSG was confirmed to inhibit the progression of apoptosis and fibrosis in HF by regulating key molecules in Hippo pathway (Figure 9).

Our data confirmed that Hippo pathway was involved in the process of myocardial apoptosis and myocardial fibrosis in HF after acute myocardial infarction. In our study, MST1/2 and P-MST1 were found to increase in marginal zone of ischemic area in HF model rats. The upstream Hippo effector MST1 that is known to induce cardiomyocyte apoptosis during the development of HF can translocate to mitochondria and phosphorylate Bcl-2, causing Bax activation and then leading to cardiomyocyte apoptosis during myocardial ischemic injury [11–13]. Meanwhile, MST1 phosphorylates and activates LATS1/2. Then, p-LATS1/2 activates P53 or phosphorylates YAP and TAZ, which can also contribute to apoptosis [14]. In this experiment, the expressions of P- LATS, P-YAP, P-TAZ, and P-53 were all elevated in HF while they were all decreased with the treatment of QSG. Together, our study demonstrated that the apoptotic processes were inhibited by QSG during the development of HF.

Cardiac fibrosis is characterized by an imbalance between extracellular matrix proteins and accumulation of extracellular matrix proteins in the cardiac interstitium and contributes to both systolic and diastolic dysfunction during the progression of HF [34–36]. In this study, administration of QSG effectively decreased the level of serum ALD, PIIINP, MMP-2, and MMP-9 that are all closely related to myocardial fibrosis in HF. In addition, transcriptomics research also confirmed the changes in extracellular matrix pathway and the regulation of QSG on it in HF. TGF-*β*, which can produce a large amount of collagen and promote mutual expression with Hippo pathway, has been considered the major contributor that can drive myocardial fibrosis [37, 38]. Due to the lack of DNA-binding domains, YAP and TAZ have to cooperate with transcription factors to stimulate gene transcription [39]. As a main downstream signal transducer of TGF-*β*1, SMAD3 is the most critical YAP/TAZ target transcription factor which can be phosphorylated by TGF-*β*1 and then promotes expression of target genes including type I and type III collagen which are the primary component of the extracellular matrix [40, 41]. Of note, a recent study reveals that myofibroblast- and cardiomyocyte-specific activation of SMAD3 has contrasting functional outcomes in healing myocardial infarction [42]. Therefore, in our study, whether the regulatory role of QSG is reflected in cardiomyocytes or fibroblasts remains to be explored in subsequent studies. CTGF, downstream target of YAP/TAZ, is another factor that can act as a bridge between Hippo and TGF-*β* pathway to induce fibrosis [43, 44]. Numerous studies show that therapeutic strategies aimed at inhibition of above profibrotic proteins are beneficial for treating HF [45–47]. The protein levels of YAP, TAZ, TGF-*β*1, SMAD3, and CTGF were all downregulated with the administration of QSG. It confirmed that the antifibrotic mechanism of QSG against HF is probably mediated by suppressing the Hippo signaling pathway.

## 5. Conclusions

QSG was proved to inhibit the progression of apoptosis and fibrosis in HF; the potential mechanism may be associated with regulating the key molecules in the Hippo pathway. It shows that the regulation of multiple targets in the Hippo pathway can achieve synergistic effects in the treatment of HF and provides insights into alternative therapies for antiapoptosis and antifibrosis after HF.

## Figures and Tables

**Table 1:** Pharmaceutical ingredients of *Qishen* granules.

Latin name	Species	Family	Part used
Radix Astragali	*Astragalus membranaceus* (Fisch.) bge.var.	Leguminosae	Roots
	*Mongholicus* (bge.) Hsiao		
Radix Salvia Miltiorrhizae	*Salvia miltiorrhiza* bge.	Labiatae	Roots
Flos Lonicerae	*Lonicera japonica* Thunb.	Caprifoliaceae	Flowers
	*Lonicera confusa* DC.		
	*Lonicera hypoglauca* Miq.		
	*Lonicera dasystyla* Rehd.		
Radix Scrophulariae	*Scrophularia ningpoensis* Hemsl.	Scrophulariaceae	Roots
Radix Aconiti Lateralis Preparata	*Aconitum carmichaeli* Debx.	Ranunculaceae	Roots
Radix Glycyrrhizae	*Glycyrrhiza uralensis* Fisch.	Leguminosae	Roots

*Note.* The ratio of these herbs was 30 : 15 : 10 : 10 : 9 : 6.

## Data Availability

The data used to support the findings of this study are available from the corresponding author upon request.

## Supplementary Materials

Supplementary Materials Table S1: the upregulated differentially expressed genes between model and sham groups. Table S2: the downregulated differentially expressed genes between model and sham groups. Table S3: the upregulated differentially expressed genes between QSG and model groups. Table S4: the downregulated differentially expressed genes between QSG and model groups.
