# Corrigendum to “Antiapoptosis and Antifibrosis Effects of *Qishen* Granules on Heart Failure Rats via Hippo Pathway”

**DOI:** 10.1155/2020/8569251

**Published:** 2020-07-01

**Authors:** Jian Zhang, Junjie Liu, Sheng Gao, Weili Lin, Pengrong Gao, Kuo Gao, Yili Zhang, Kangjia Du, Xiaomin Yang, Wei Wang, Ruixin Zhu, Yong Wang

**Affiliations:** ^1^School of Life Science, Beijing University of Chinese Medicine, Beijing 100029, China; ^2^Dongzhimen Hospital, Beijing University of Chinese Medicine, Beijing 100700, China; ^3^School of Life Sciences and Technology, Tongji University, Shanghai 200092, China; ^4^School of Traditional Chinese Medicine, Beijing University of Chinese Medicine, Beijing 100029, China

In the article titled “Antiapoptosis and Antifibrosis Effects of *Qishen* Granules on Heart Failure Rats via Hippo Pathway” [1], there was an error in Section 2.5 “Measurement of Serum Biochemical Markers” of the article body. Section 2.5 “Measurement of Serum Biochemical Markers” should be updated as follows:

**Measurement of Serum Biochemical Markers:**

At the end of the cardiac function examination, rats were anesthetized with 1% pentobarbital sodium (50 mg/kg) by intraperitoneal injection, and blood samples were collected from the abdominal aorta and centrifuged at 1000 ×g for 20 min to obtain serum.Serum levels of ALD and PIIINP were measured by radioimmunoassay. BNP, ANP, MMP-2, and MMP-9 were detected by enzyme-linked immunosorbent assay following the instructions of kits (Fanke Biological Technology Company, Shanghai, China).
